# Nonlinearities in Fringe-Counting Compact Michelson Interferometers

**DOI:** 10.3390/s23177526

**Published:** 2023-08-30

**Authors:** Jiri Smetana, Chiara Di Fronzo, Anthony Amorosi, Denis Martynov

**Affiliations:** 1 Institute for Gravitational Wave Astronomy, School of Physics and Astronomy, University of Birmingham, Birmingham B15 2TT, UK; d.martynov@bham.ac.uk; 2 Precision Mechatronics Laboratory, A&M Department, Université de Liège, Allé de la Découverte 9 B52/Quartier Polytec 1, B-4000 Liège, Belgium; cdifronzo@uliege.be (C.D.F.); anthony.amorosi@uliege.be (A.A.)

**Keywords:** displacement sensor, Michelson interferometer, nonlinearity

## Abstract

Compact Michelson interferometers are well positioned to replace existing displacement sensors in the readout of seismometers and suspension systems, such as those used in contemporary gravitational-wave detectors. Here, we continue our previous investigation of a customised compact displacement sensor built by SmarAct that operates on the principle of deep frequency modulation. The focus of this paper is the linearity of this device and its subsequent impact on sensitivity. We show the three primary sources of nonlinearity that arise in the sensor: residual ellipticity, intrinsic distortion of the Lissajous figure, and distortion caused by exceeding the velocity limit imposed by the demodulation algorithm. We verify the theoretical models through an experimental demonstration, where we show the detrimental impact that these nonlinear effects have on device sensitivity. Finally, we simulate the effect that these nonlinearities are likely to have if implemented in the readout of the Advanced LIGO suspensions and show that the noise from nonlinearities should not dominate across the key sub-10 Hz frequency band.

## 1. Introduction

Interferometry sits at the forefront of high-precision displacement measurement. This technique sees use in a wide range of contexts concerned with the detection of weak signals, which originate from the minute scale of quantum mechanics through to the grand scale of astrophysical phenomena. Amongst the most notable interferometric devices are the contemporary gravitational-wave (GW) detectors at the Advanced Laser Interferometer Gravitational-Wave Observatory (LIGO) [1] and Advanced Virgo [2], which are capable of $2\times 10^{-20}m/\sqrt{\mathrm{Hz}}$ precision in the peak sensitivity band around 100 Hz [3].

Although specialised detector facilities, such as GW detectors, can span kilometre scales, compact interferometric devices on the centimetre scale can be traced back to 1972 [4]. Since then, the sensitivity of such devices has continued to improve, with the Laser Interferometer Space Antenna (LISA) Pathfinder [5] mission showing the versatility of interferometry in space-based applications. Advances in miniaturisation of optical and laser components have led to a range of compact devices that offer excellent sensitivity, as we outline below. The key to the impressive sensitivity an interferometer is its extremely sharp response to small displacements, with the sensor’s full range spanning the scale of the wavelength of light used. For example, the well-studied, sinusoidal response of a Michelson interferometer (e.g., [6]) operated with a typical 1064 nm laser covers the full signal range over a narrow span of just 255 nm.

A thorough overview of notable interferometric sensors can be found in Ref. [7]. In this paper, we focus principally on the performance of our custom-designed compact Michelson-type sensor built by SmarAct and analysed previously in Ref. [8]. The nominal subpicometre sensitivity that was achieved is useful across a range of applications (see Ref. [8] and references therein), but we particularly focus on its utility in the sensing of quiet suspension systems, namely the quadruple suspension [9] of Advanced LIGO detectors. Current suspensions are sensed with Birmingham Optical Sensor and Electromagnetic Motor (BOSEM) shadow sensors [10], which utilise an optical sensing scheme, albeit not an interferometric one. As argued in detail in Ref. [11], the low-frequency band (5–30 Hz) is limited by the injection of noise from angular control loops (also shown in Ref. [3]), which can ultimately be traced back to the limiting sensitivity of existing shadow sensors. Sensitivity improvements in this detection band are essential in enhancing early-warning systems [12,13] and expanding the range of detected GW sources towards intermediate-mass black holes [14].

Existing GW detectors use a range of inertial and displacement sensors to improve the stability and provide active isolation and readout control of their suspension systems. However, the detectors stand to benefit from further improvements in inertial sensors. A notable way to improve these devices is with better sensors, with current devices being broadly limited at low frequencies by their readout noise. Interferometric sensors are well poised to address this problem, with one example, the Homodyne Quadrature Interferometer (HoQI) [15], already demonstrating an improvement in the low-frequency sensitivity of a commercial geophone [16]. Recent high-precision devices, such as beam rotation sensors (BRS) [17,18] and six-degree-of-freedom (6DoF) inertial sensors [19,20], use interferometric sensing to achieve advanced performance. We expect that our sensor will be used in the future testing of the compact 6D inertial sensors [21], an evolution of the previous 6D design.

The path towards improved sensitivity in the key frequency band lies in the development of better sensors. The SmarAct interferometric sensor represents a good candidate to achieve this. However, to satisfy the requirements laid out in Ref. [11], it will be important to reach the full sensitivity level demonstrated in Ref. [8]. This is only possible if the performance of the sensor is not degraded once placed into the real environment rather than the typical ‘null measurement’ setup used in its noise characterisation. Of particular interest to us is the impact of the sensor’s linearity on the realistic sensitivity limit.

In this paper, we investigate the impact of nonlinear noise in Michelson-type interferometric displacement sensors when placed in applications where the root mean square (RMS) displacement exceeds the effective RMS displacement of the sensor’s own noise by multiple orders of magnitude. The range of a simple Michelson interferometer is already narrow, and its sinusoidal response means that the usable linear region yields an even smaller operating range. A number of different techniques exist in the literature [7] to extend this range through the use of multiple phase-offset readout channels, which allow for a linearised estimate of the displacement. Our particular readout scheme, which is based around the principle of deep frequency modulation (DFM) [22,23], is discussed in more detail in Section 2. This readout scheme theoretically fully linearises the displacement and, with the use of a phase-unwrapping algorithm (known in this context as fringe counting) [24,25], can extend the range of the interferometer over many multiples of the free spectral range (FSR). However, the linearisation algorithm can suffer from limitations that, in practice, lead to imperfect linearisation of the signal and the injection of periodic nonlinear error into the readout. The mechanisms of nonlinear coupling are analysed in Section 2.3, Section 2.4 and Section 2.5, with a theoretical framework laid out for modelling these nonlinearities in a real-world situation. The theoretical model is compared to measured data from an experimental scheme described in Section 3. This theoretical model is applied to a simulation of the nonlinear noise performance in suspension sensing within LIGO vacuum chambers in Section 4.

## 2. Modelling Nonlinearities

**Figure 1:**
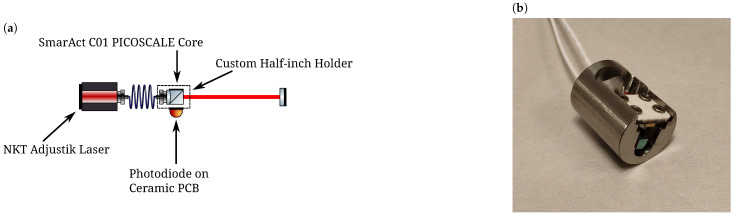
Schematic (**a**) and photo (**b**) of the custom-designed sensing head manufactured by SmarAct that is used throughout this work. The sensing head is embedded within the same readout structure as shown in Figure 1 of Ref. [8].

The sensor scheme is based around a custom-designed optomechanical assembly derived from the SmarAct C01 PICOSCALE sensing head. The sensor shown in Figure 1 consists of a simple Michelson interferometer with an open port along one of the typical interferometer arms and a high-reflectivity coating applied to one face of the central beam splitter cube to act as the reference arm. This minimalist design leads to a highly compact sensor and mitigates losses from additional optical components. The simplicity of the design, a key aspect of the robust and sensitive scheme we investigated in Ref. [8], results in a comparatively greater level of complexity in the algorithm employed in the phase extraction scheme.

We make use of the DFM technique described in depth in Refs. [22,23] that has seen growing applications in interferometric displacement measurement [26,27,28]. The scheme begins with a modulation of the laser frequency by any number of standard techniques—in our case, control of the laser cavity piezoelectric transducer (PZT). Solving for the signal measured by a photodiode at the output of an unbalanced Michelson interferometer, we obtain the following simple relation:

(1) $$P\left(t\right)=A[1+C\cos\left(\phi \left(t\right)+m\cos\left(\omega_{m}t\right)\right)],$$

where *A* is the signal scale factor (akin to amplitude); *C* is the fringe contrast, a value in the range 0–1 corresponding to the level of mode matching of the interfering beams; ϕ is the additional microscopic arm phase accumulated within the measurement arm of the interferometer; *m* is the modulation index; and $\omega_{m}$ is the modulation angular frequency. The arm phase can be straightforwardly related to the mirror displacement via x=ϕλ/(4π). The modulation index can be intuitively written as $m=4\pi A_{m}\Delta L/c$, where $A_{m}$ is the modulation depth in term of frequency such that the time-dependent laser frequency can be written as $f\left(t\right)=f_{0}+A_{m}\cos\left(\omega_{m}t\right)$, and ΔL is the difference in length between the reference arm and the measurement arm of the interferometer. This result is commonly processed further in the limit of m≪1, where small-angle approximations are appropriate and lead to a host of widespread uses, such as in cavity locking schemes (e.g., Pound–Drever–Hall locking [29]). It is beneficial to consider the signal obtained in Equation (1) as a Fourier series decomposition in terms of the harmonics of $\omega_{m}$, which is given by

(2) $$P\left(t\right)=P_{0}+\sum_{n=1}^{\infty }2CAJ_{n}\left(m\right)\cos\left(\phi +n\pi /2\right)\cos\left(n\omega_{m}t\right),$$

where $J_{n}$ is the *n*th-order Bessel function of the first kind, and $P_{0}=A\left(1+CJ_{0}\left(m\right)\cos\left(\phi \right)\right)$. While we may recover the typical small-angle approximation by considering only the n=1 term, we operate the system beyond the small *m* limit, which necessarily extends the scheme to include the higher-order terms. This is important for us, as it gives us access to multiple signals with a cyclical sinusoidal dependence on the arm phase (ϕ). If we isolate the individual harmonic terms from the sum in Equation (3), we obtain these multiple cyclical terms—the same effect as reading out a sequence of interferometers, each offset from the previous sequence by a quarter period but with the added benefit of sharing some noise sources in common. From these, we can construct a linear estimator of the phase.

Our particular scheme relies on a technique of demodulation at multiple harmonics of $\omega_{m}$. If we multiply the raw signal by cos(kωt) for integer vales of *k* and filter out the beat signals above DC with an appropriate low-pass filter, we can write the *k*th demodulated harmonic as

(3) $$S_{k}=CAJ_{k}\left(m\right)\cos\left(\phi +k\pi /2\right).$$

Assuming we have a stable scheme such that *A*, *C*, and *m* remain constant, the simplest way to proceed is to take a pair of $S_{k}$ signals of different parity, for example, from the k=1 and k=2 demodulation, and construct an elliptical Lissajous figure where the angular coordinate of a point along the Lissajous curve at any given time corresponds to the arm phase (ϕ).

If we proceed with the first two harmonics, we obtain the arm phase by applying the four-quadrant arc tangent through

(4) $$\phi =\arctan\left(\alpha \frac{S_{1}}{S_{2}}\right),$$

where α is a normalisation factor that compensates for the unequal sideband amplitudes. This value is determined through a sweep over at least one FSR, where we subsequently estimate the value of α from the ratio of the amplitudes of $S_{1}$ and $S_{2}$. Nominally, this value should be exactly $\alpha =J_{2}\left(m\right)/J_{1}\left(m\right)$ and, in our case, is equal to unity, as we tune *m* such that $J_{1}\left(m\right)=J_{2}\left(m\right)$.

The optical and digital layout of our scheme can be found in Figure 1 of Ref. [8]. The optical and electrical system presented in this work is near-identical to that used in Ref. [8]. We inject a frequency modulation with $A_{m}=0.4$ GHz amplitude at a frequency of $\omega_{m}=2\pi \times 700$ Hz into the sensing head. The measurement mirror is placed at a distance of 15 cm from the tip of the sensing head. Due to the redesigned sensing head, we detect the interference at the previously unused output port as shown in Figure 1a.

The new photodiode has a slightly higher responsivity of $1.1\;AW^{-1}$, but the signal is passed through the same 68 kΩ transimpedance amplifier to the data acquisition system. As in our previous work, we make use of the real-time data processing architecture derived from the control and design system (CDS) used in Advanced LIGO. This consists of a 20 V peak-to-peak analogue-to-digital converted and a 10 V peak-to-peak digital-to-analogue converter digitised at 64 kHz and downsampled to 32 kHz for processing.

### 2.1. Nonlinearities and Range

Nonlinearities intuitively grow in severity with an increase in the range of measurement. We consider our sensor to be ‘long-range’ according to two specific criteria. First, the sensor can perform linear measurements over multiples of the FSR. This is a general property of a fringe-counting interferometer but, nonetheless, distinguishes our sensor from the non-fringe-counting interferometers that are more common in the field.

Secondly, we consider the sensor long-range due to the comparatively large span of ‘null’ positions at which it can be set. In the experimental demonstration, we tune the frequency modulation depth so that we balance the power in the first two harmonics. This is a restriction we impose in order to maintain stationary noise over the full range of operation. However, this condition can be achieved over a range of different test-mass separations as long as the correct depth of frequency modulation can be reached with the given laser.

With our particular laser, we consider the minimum test-mass distance to be 1.6 cm, corresponding to a modulation amplitude of 4 GHz—comfortably within reach of many other existing lasers. The modulation requirements are relaxed with increasing distance; therefore, the test mass can, in principle, be placed far from the sensor. Other complications, such as sensitivity to test-mass angular motion, diminishing fringe contrast, and growing coupling to laser frequency noise, impose practical limitations on the maximum distance. The maximum distance is therefore application-dependent. We find that the fringe contrast drops below 0.5 above a distance of 20 cm, which we choose as our criterion for maximum separation.

A key feature of our sensing scheme is the overall simplicity of the optical components coupled with the simplest implementation of the DFM scheme possible. Of course, the strength of the DFM scheme lies in the ability to extract information from an arbitrary number of harmonics, limited only by the modulation capabilities of the laser. In Ref. [8], we demonstrated phase extraction from multiple harmonics via the subtraction of common phase noise between two sensing heads, where the algorithm was applied on the first and second harmonics for one sensor and the second and third harmonics for the other sensor. Significantly more complex algorithms exist, which make use of many more harmonics and simultaneously extract all of the parameters from Equation (1), notably including the modulation index (*m*). These algorithms (examples provided in Section 2.2) unlock the full potential of the DFM technique but, as a consequence, are substantially more complex and significantly increase the stringency of the requirements in terms of the laser’s performance.

### 2.2. Sources of Nonlinearity

Nonlinear effects in optical interferometry have been analysed in the past and are known to lead to a periodic error on the order of a few nanometres [30,31]. These effects commonly arise from cross talk between the two nominally orthogonal signals, for example, due to imperfections in the polarisation optics in interferometers that utilise linearly polarised states of light, such as the HoQI [15].

In our case, the signals channels are well isolated from each other, as their orthogonality is ensured by a rigid mathematical relation shown in Equation (2) and not dependent on the quality/alignment of optical components. This is in contrast with interferometers that use multiple polarisations and multiple photodiodes, and care must be taken to ensure that all photodiodes are simultaneously aligned and that the polarisations are well separated. However, other types of common nonlinearities can still occur, for example, the coupling of ghost beams into the readout port [32,33,34]. These nonlinear sources are important to consider and are certainly present to an extent within our sensor.

Ghost beam effects are mitigated by the relatively few optical surfaces involved in the interferometric path. Furthermore, the principal ghost beams occur through interactions between surfaces of the central beam cube, where drifts in the ghost beam phase arise due to thermal expansion of the beam cube. These effects are naturally mitigated by the cube’s small volume (side length of 2 mm) and the good thermal properties of glass.

Consequently, the dominant ghost beams arise from higher-order reflections, which can pick up significant phase fluctuations through propagation outside of the beam cube, such as the reflections from the measurement port’s antireflective coating causing the beam to pick up the measurement arm’s phase twice. The initial sensing head design measured the interference on the return path down the fibre through the laser input port, separated from the incoming field by a fibre circulator. This was deemed undesirable due to the potential coupling of additional noise from fibre backscatter and stress relaxations through parasitic interference. With the redesign, we somewhat mitigate this issue by propagating the light from the beam splitter to the photodiode through free space, although this has not eliminated the problem entirely. Ultimately, ghost beams can be problematic for high-precision applications, and their presence in our sensor is being investigated.

The DFM technique leads to a host of nonlinearities that are specific to it. One such effect arises from the residual amplitude modulation that inevitably accompanies the desired frequency modulation. This is easily understandable in the context of a distributed feedback (DFB) laser, where frequency modulation is achieved by a modulation of the supply current, which is also directly responsible for controlling the laser power. Even in cases where the laser power and frequency are controlled by separate means, the laser cavity PZT in our case, there remains a non-negligible coupling with amplitude in the frequency modulation channel. In such a case, the power amplitude term (*A*) in Equation (1) contains a dynamic component at a given frequency ($\omega_{m}$), which primarily contributes an additional term to the power in the n=1 harmonic and is insensitive to the arm phase.

This leads to an offset and rotation in the demodulated signals in Equation (3), which ultimately leads to a distortion of the Lissajous figure, a nonlinear effect. The amplitude modulation index is entirely governed by the modulation at the laser, while the frequency modulation index is expressed by the combined effect of the depth of frequency modulation ($A_{m}$) and arm imbalance (ΔL). Therefore, it is possible to scale the relative amount of residual amplitude modulation by setting up the sensor at different positions with different ΔL values and tuning the laser to maintain the same frequency modulation index. Through a series of such measurements, we find that residual amplitude modulation does not have a dominant impact on either the noise performance or linearity within our range of operation. It is possible that for significantly deeper modulation indices beyond our considerations, the residual amplitude modulation may make the first harmonic unusable.

Fluctuations in the value of the modulation index are yet another source of nonlinearity that arises in the simplified DFM readout scheme that we implement. Due to the use of only two harmonics for signal extraction, we can only make a real-time measurement of the arm phase parameter and assume that all remaining parameters remain constant. Even if we relax the condition that $J_{1}=J_{2}$ at the expense of noise stationarity, we still require that the modulation index remain constant or fluctuates little between periodic corrections. If this is not the case, then the amplitude of the terms in Equation (3) fluctuates over time, causing dynamic distortions in the Lissajous shape, introducing nonlinear effects. The two main causes of a fluctuating modulation index are changes in ΔL and $A_{m}$. We assess the maximum extent of this type of nonlinearity in Section 2.4.

The final source of non-algorithmic nonlinearity we consider is the nonlinearity of the frequency modulation. The modulation is generated by the PZT using a linear drive. However, piezoelectric actuators are known to exhibit a nonlinear response, particularly hysteresis. This effect was investigated in the context of DFM interferometry in Ref. [26] and our laser’s PZT linearity was characterised in our previous work [8]. We found that when we inject a high-amplitude slow oscillation to the laser PZT to simulate a large sinusoidal displacement, the PZT nonlinearity dominates over the residual nonlinearity of the phase-extraction algorithm by an order of magnitude. Therefore, it is essential that we avoid large offsets in the modulation drive signal, which imposes a significant practical limit on the range of our frequency stabilisation servo implemented in Ref. [8].

Depending on the sources of nonlinearity, different approaches to improving the linearity and range exist in the literature. The suppression of ghost beams was tackled in Ref. [34] through a hardware solution involving a more complex optical layout and expanding the number of readout ports. Linearity can also be improved with software solutions, i.e., implementing more advanced algorithms often run on a field-programmable gate array (FPGA). Some examples include the use of a more complicated demodulation waveform [35] or fitting to multiple complex harmonic amplitudes in the frequency domain [22,36]. In these cases, the inclusion of additional information by considering more than the minimum number of harmonics improves the linearity and increases the dynamic range of the sensor. This is particularly the consequence of allowing for the modulation index to become a dynamic, fitted quantity rather than an assumed constant.

We find that the dominant sources of nonlinearity arise from the particulars of the digital processing within the DFM scheme and can be traced back to the fidelity of the modulation–demodulation procedure, the accurate constraint and fitting of the parameters in Equation (3), and the limited bandwidth of the sensor. Following common wisdom, we expect that nonlinearities increase disproportionately with growing signal RMS. The question is, however, whether these nonlinear effects can reduce the signal-to-noise ratio (SNR) below unity for a realistic RMS displacement or before reaching other natural constraints, such as the velocity limit imposed by the fringe-counting algorithm.

We consider a situation where we reach the upper realistic limit of RMS displacement within the context of suspension or seismometer sensing under ambient seismic conditions (order of 1 μm). We further stipulate that the RMS is concentrated in a high-amplitude region of signal within a limited frequency band, with signal outside of this band settling at a much lower level by several orders of magnitude. Our illustrative example is a flat band-limited ‘tophat’-shaped spectral density function. Due to the nonlinear processes leading to a beat between pairs of frequencies across the whole spectrum, it becomes possible for the high-amplitude signal to down- and upconvert to other frequencies and thus swamp the true weak signals in the quiet regions of the spectrum. We investigate such a scenario, where the additional ‘nonlinear noise’ generates a new and degraded noise performance, which prevents our device from reaching its nominal subpicometre sensitivity.

This scenario is not entirely academic, as it shares a practical similarity with the signals handled in inertial sensing devices. The response of a conventional mass-on-a-spring inertial sensor begins to substantially decrease towards DC, below the mass-spring resonant frequency. This feature can be found in commercial seismometers such as the Trillium T240, and even more so in the velocity readout (velocity readout multiplies the response to displacement in the frequency domain by an additional factor of ω, further reducing the response towards DC.) of geophones such as the Sercel L-4C, as well as in custom, precise angular sensors, such as the BRS and multi-degree-of-freedom sensors such as 6D. In these applications, we are searching for very weak readout signals at low frequencies, while the high response at and above the resonance leads to a potentially large signal RMS.

### 2.3. Nonlinearities from Ellipticity

Our phase extraction algorithm takes multiple nonlinear functions of ϕ (specifically sinusoidal functions) and combines them to form a linearised readout. This algorithm relies on the correct knowledge or fitting of the ellipse parameters, which are used to circularise the ellipse for use with the four-quadrant arc tangent function. Therefore, if there is a mismatch between the ellipse parameters, some residual ellipticity to the Lissajous figure remains, which translates into a systematic nonlinear error.

**Figure 2:**
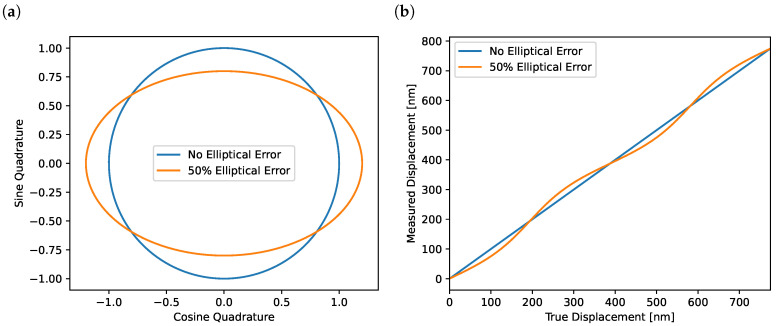
Effect of elliptical error on the estimation of the arm phase (test mass displacement). (**a**) An incorrectly circularised Lissajous figure with a 50% residual elliptical error in comparison to a properly circularised Lissajous figure. Panel (**b**) Corresponding periodic nonlinear error caused by this residual ellipticity. In comparison, the perfectly circularised Lissajous figure leads to a perfectly linear estimator. The 50% error was chosen for visual clarity only and is significantly larger than what is typically observed in our system.

We define a quantity, i.e., the elliptical error (δ) expressed as the fractional difference between the semimajor and semiminor axes such that the semimajor axis is expressed as a=(1+δ)b for a semiminor axis of size *b*. The visual effect of the elliptical error on the Lissajous shape is shown in Figure 2a, with the corresponding periodic error on the displacement readout shown in Figure 2b. Through a Taylor expansion in δ (we can assume that in all reasonable scenarios, δ≪1), we find that the phase estimator ($\bar{\phi }$) is related to the true arm phase approximately through

(5) $$\bar{\phi }\approx \phi +\frac{\delta }{2}\sin\left(2\phi \right),$$

where the second term represents the first-order contribution to the periodic error that is generated as a result of the ellipticity.

We cannot proceed further in deriving a general spectral density equation for nonlinear noise. However, we may use this as the basis of the time-domain simulations of the nonlinear effects, which allow us to model and predict the nonlinear impact in particular applications. Additionally, due to the limited range of the sine function, we can estimate the maximum noise floor that can be generated by this nonlinearity.

Within the limit of broadband, high-RMS displacement, which we define as a situation where the fluctuation in ϕ exceeds unity, we consider sin(2ϕ) to behave as a generator of random values within the interval of [−1, 1]. Therefore, the variance of this term is within a factor of a few below unity; we adopt a representative value of 1/3, corresponding to a uniform distribution. The power spectral density (PSD) that corresponds to this variance is broadened out as the original displacement spectrum saturates the sine function and spreads to other frequencies, leading to the approximate PSD of the sine term, i.e., $S_{\sin}\approx 1/3\gamma_{eff}$, where $\gamma_{eff}$ is the effective bandwidth of the frequency-broadened signal. This bandwidth is highly variable based on the exact shape and RMS of the signal. However, as the noise is only ever broadened, the highest noise level that can be achieved occurs at $\gamma_{eff}=\omega_{hi}$—the upper edge of the frequency band of the original displacement signal. Thus, our order-of-magnitude estimate for the maximum nonlinear noise PSD is given by

(6) $$S_{x}^{\max}=\frac{\lambda^{2}\delta^{2}}{192\pi^{2}\omega_{hi}}.$$

This equation only provides the maximal noise floor in the case of a sufficiently broadband signal and is valid in the frequency band below $\gamma_{eff}$. As such, this noise level can be exceeded where nonlinear upconversion occurs, particularly in the case of tightly localised resonances in the power spectrum that lead to the presence of prominent peaks at higher harmonic frequencies.

This nonlinearity can arise due to a poor estimate of the ellipse parameters. To prevent this issue, it is possible to perform a slow sweep over the laser frequency, provided the laser frequency actuator has the range to sweep over at least one full FSR. We can fit to the resulting ellipse, assuming the ellipse parameters do not change over time. In reality, a number of effects can generate a drift in the ellipse parameters, such as nonlinearity in the modulation drive, timing jitter causing drifts in the demodulation phases, and a relative shift between the frequency modulation and residual amplitude modulation waveforms. We find that maintaining elliptical error at or below δ=0.01 over a two-week period of operation is feasible. The error settled to this value over approximately a one-day timescale and showed no signs of further degradation, suggesting that the linearity of the sensor can be maintained at this value beyond the two-week measurement period.

Importantly, we observe that the predicted maximum nonlinear noise floor is reached at an RMS just within the range of a single fringe. This means that to demonstrate the limiting nonlinear noise in the sensor, it is not necessary to operate the sensor under high dynamics (above 1 um RMS). Eventually, under sufficiently high displacement, other nonlinearities, most prominently the changing modulation index due to large ΔL fluctuations, begin to dominate; then, the linearity begins to degrade again with increased motion, as expected. Assuming we can maintain a residual elliptical error of 1%, we can tolerate a displacement range between 0.14 mm (for 1.6 cm arm mismatch) and 1.8 mm (for 20 cm arm mismatch) before the modulation index change begins to dominate the nonlinearity. This level of displacement is above the range requirements for our target applications of suspension and seismometer sensing (typically on the scale of microns or less) and thus beyond the appropriate limit of our sensor and algorithm design.

### 2.4. Nonlinearities from Nonellipticity

The extension of the above treatment of ellipticity leads us to consider nonlinearities that occur due to a departure from an elliptical Lissajous shape altogether. According to our long-term observations, the sensor produces a high-fidelity elliptical Lissajous figure, and in most situations, the nonlinearities tend to be dominated by poorly fitted ellipse parameters. However, even with proper ellipse fitting in post processing, we find residual periodic error in the phase readout, which suggests that the Lissajous figure is not entirely elliptical.

This departure of the Lissajous figure from a simple elliptical shape was previously observed in Ref. [8], where we showed the nonlinear error in the displacement readout using a long-range (∼100 FSR) scan over the laser frequency using the temperature set point. We revisit this result here, with a closer look at the amplitude and period of the error.

**Figure 3:**
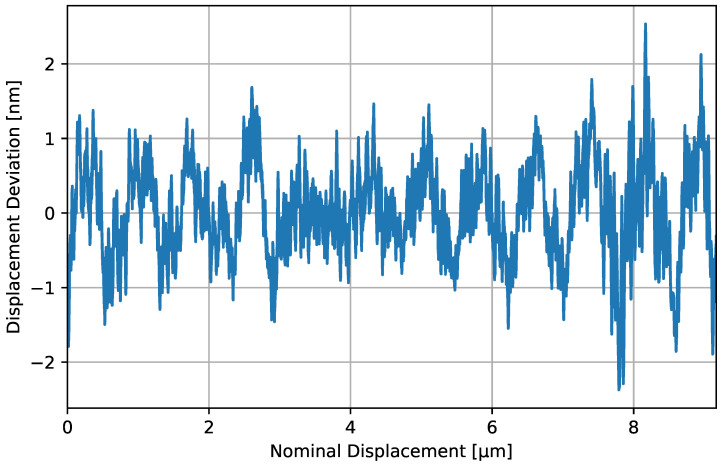
Nonlinear deviation of sensor readout from the ‘true’ inferred displacement. The x axis is calibrated for displacement by taking the displacement time series and fitting to the slow variations (not due to sensor nonlinearity) by a ninth-order polynomial.

We sweep over the effective displacement by slowly actuating on the laser wavelength. A benefit of this method is that the modulation index does not change, as it would if the sweep were performed over true displacement, introducing the nonlinearity associated with a dynamically changing Lissajous figure due to a non-constant modulation index. This is achieved by setting the laser wavelength from one extreme to another (sweeping over a total wavelength span of 0.9 nm) and allowing the built-in temperature servo to shift the wavelength to the new set point. We isolate a region containing approximately 10 fringes in the middle of the sweep where the wavelength was swept through approximately linearly in time. We linearise the readout using an elliptical fit in post processing, then remove the underlying, slowly varying nonlinearity of the sweep using a ninth-order polynomial fit. As the period of the sensor nonlinearities is much shorter than any trend in the sweep, we can remove these trends without also fitting to the periodic error. The result presented in Figure 3 shows the deviation of the measured displacement signal from the fitted ‘nominal’ displacement over the swept region. The nominal displacement is given by the estimated true phase (calibrated for displacement) covered over the wavelength sweep, which is derived from the ninth-order polynomial fit to the time series.

We note that the nonlinear deviation cannot be described by a simple function. However, by observing the power spectrum of the deviation in Figure 3, we note the existence of several peaks, suggesting there is a periodicity to the nonlinear deviation. The most prominent peak occurs at a spatial period of approximately 610 nm. With a periodic error, we can adopt the same approach as we did in the analysis of Equation (5) and assign an *effective* elliptical error ($\delta_{eff}$), which should provide an estimate of the nonlinear impact to within a factor of a few. Taking the amplitude of the nonelliptical deviation to be 1.2 nm, this yields a $\delta_{eff}$ of around 2%. As this nonlinearity arises through (as yet unknown) processes that distort the Lissajous figure away from an ideal ellipse, it is not clear how to suppress this nonlinearity further. Therefore, for now, this imposes a hard limit on the linearity of the sensor. It is certainly possible that this nonlinear effect arises due to the limitations of the physical sensing head design, such as the remnant fibre backscatter and ghost beam couplings. These effects will be investigated and addressed in future development of the sensor design.

### 2.5. Nonlinearities from Demodulation

The final nonlinearity we consider comes from the upconversion of signal frequencies through the sine function interacting with the finite bandwidth of the sensor. Our algorithm relies on the low passing of the demodulated signals. If we consider a purely sinusoidal displacement at some arbitrary frequency (Ω), leading to an arm-phase fluctuation given by ϕ=Asin(Ωt), our demodulated signals are proportional to sin(Asin(Ωt)) (replacing the outer sine with cosine for the even harmonics).

This is a familiar equation within our setup and is of the type already encountered in Equation (1). Thus, the demodulated signal can be written as an infinite series with terms proportional to $J_{n}\left(A\right)\sin\left(n\Omega t\right)$ for integer *n*. We can still reconstruct the signal with sufficient accuracy only considering terms up to a particular order (*n*). To determine the maximum order that must be included, we make use of the fact that the $J_{n}\left(x\right)$ are diminishingly small for $x\ll x_{m}$, the location of their first maximum. We approximation that for a particular order, the location of the first maximum is well approximated by the value of the order itself [37]. Therefore, we conclude that the critical order is given by n≈A, and we can neglect all orders where n≫A.

In a practical sense, this means that the maximum frequency that our original signal is significantly upconverted to is given by approximately AΩ. This is consistent with the intuitive argument that the frequency of the outer sinusoid is given by the phase velocity ($\dot{\phi }$) and $A\Omega ={\dot{\phi }}_{\max}$. Taking this intuitive argument further, we propose that for any arbitrary displacement, the corresponding ${\dot{\phi }}_{\max}$ must be significantly below the cutoff frequency of the low-pass filter. In a sense, the finite bandwidth of the sensor ($\gamma_{s}$) imposes a velocity limit on the sensor application, as expressed by

(7) $$v_{\max}\ll \frac{\gamma_{s}\lambda }{4\pi }.$$

The first constraint on $\gamma_{s}$ is the low-pass filter cutoff frequency. However, this frequency cannot be increased arbitrarily, as when ${\dot{\phi }}_{\max}$ exceeds $\omega_{m}/2$, the signal side bands around the adjacent harmonics leak into the measurement band of their neighbouring harmonics and corrupt the signal there. Therefore, the sensor bandwidth is ultimately limited to $\gamma_{s}\leq \omega_{m}/2$. Taking this limit for our setup, we obtain an absolute velocity limit of around 50 $\mu m\;s^{-1}$. This bandwidth-imposed limit can be raised by increasing the modulation frequency. To enable this, we would require a faster sampling and data processing architecture. More advanced DFM algorithms have already been demonstrated on FPGAs [22,35,36], which means that a solution is readily available. However, we chose to deploy our algorithm on the CDS architecture, as this allows our system to be easily integrated into the existing LIGO infrastructure.

## 3. Sensitivity Degradation in a High-RMS Application

**Figure 4:**
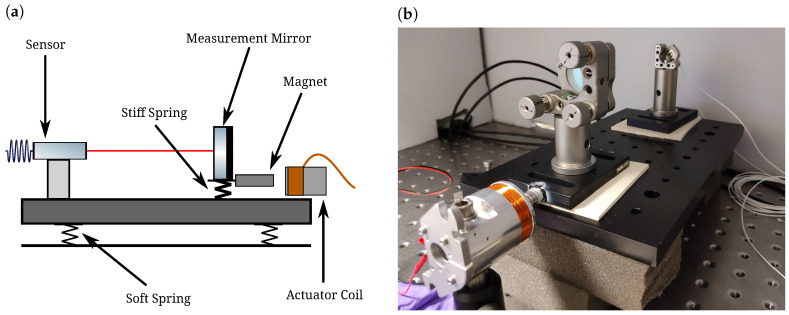
Adjusted layout of the experimental setup originally shown in Figure 1 of Ref. [8]. (**a**) Arrangement of the sensor, mirror, and actuator, as well as the effective springs formed by the foam blocks and rubber pads. (**b**) Photo of the setup inside an acoustically isolating box.

We experimentally demonstrate the nonlinear degradation of sensitivity by driving the measurement mirror with a known, high-RMS signal and observing the sensor’s resulting spectrum. We make use of a moving magnet actuator, specifically a BOSEM shadow sensor [10] with the sensing components and circuitry removed. The coil resistance is 41.4 Ω, with an inductance of 17.8 hLmH. The layout can be found in Figure 4. The sensing scheme and physical layout are identical to the scheme in Ref. [8] and shown in Figure 1 therein, except for the additions specified below.

We must isolate the sensor from environmental disturbances, such as seismic and acoustic couplings, as these generate uncontrolled and dominant sources of noise. To achieve this, we set up the mirror-sensor system in an acoustically isolated box on a common base plate placed on foam blocks for further vibration isolation. As the sensor only measures the relative displacement between itself and the mirror, the vibrational coupling to the readout can be suppressed by many orders of magnitude. In a departure from the setup described in Ref. [8], we isolate the measurement mirror from the common base plate with a rubber pad (stiffer than the foam blocks) to allow some compliance with differential motion. We actuate with the coil on a stack of three RS Pro neodymium magnets (stock number 219-2231) attached to the mirror base, which allows for some residual differential drive, although most of the driven displacement remains common.

We use a ThorLabs LDC 205 C laser diode driver to provide the coil drive current. We monitor this drive current using the built-in control port of the current driver. This is essential, as we find that the current driver cannot naturally drive inductance linearly, which we verified by comparing the linearity of the coil drive against the linearity of driving an equivalent resistor. This actuator nonlinearity dominates over the nonlinearity of the sensor and therefore must be suppressed. We notice an even stronger nonlinearity when using a mirror-mounted PZT, which leads us to discount this otherwise much simpler actuation scheme. To improve the actuator linearity, we design and implement a feedback system where we subtract the measured drive current from the desired setpoint drive to form an error signal that controls the current driver in-loop. This scheme suppresses the subkilohertz nonlinear noise by around two orders of magnitude compared to the free-running performance.

We drive the current with uniform white noise, which is then band-limited with an eighth-order elliptical band-pass filter to produce a large, flat signal spectrum confined to a sharply cut off frequency window. With this scheme, we tune the displacement RMS to around 0.2 μm and three orders of magnitude higher spectral density in the signal window than the residual noise at frequencies below the signal band. This RMS value is chosen to maximise the algorithmic nonlinearities, as motivated in Section 5. A higher RMS leads to the nonlinear error spreading over a broader frequency band, leading to a lower noise level. This RMS is also sufficiently low that the displacement does not significantly change the modulation index (around 1 ppm), so does not introduce a further source of nonlinearity. We are not interested in testing these nonlinearities here, as they occur beyond the target range of our sensor’s applications.

We drive the signal injection digitally and shape this drive using digital filters using the same CDS architecture as that used to read out the sensor. Therefore, we can freely shift the amplitude and frequency band of the signal to investigate the different levels of nonlinear noise that appear at low frequencies. We also simulate the sensor response in the time domain to compare the measured noise with the nonlinear noise models derived in Section 2.

**Figure 5:**
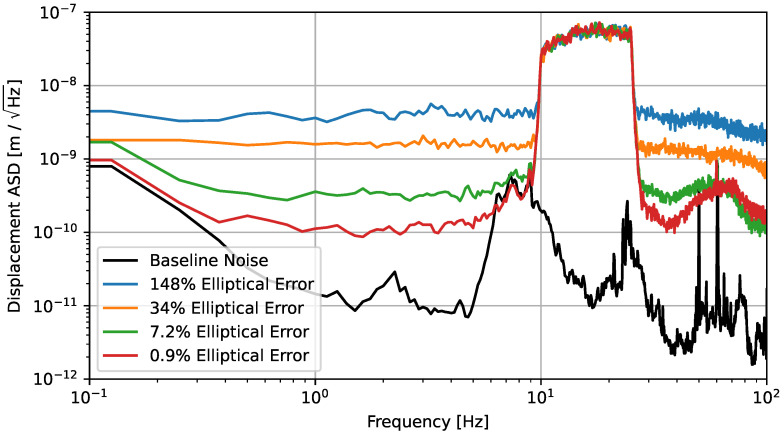

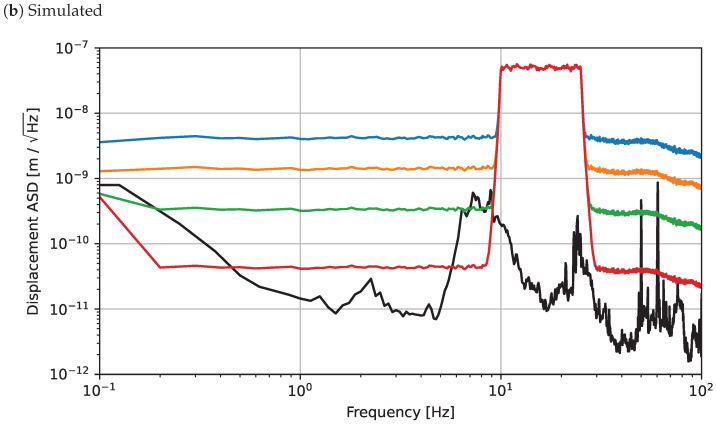
Comparison between measured and simulated nonlinearities during a controlled and tightly band-limited injection of displacement noise using a coil-magnet actuator. The simulated spectra show good agreement with measurement for high values of elliptical error, which supports the analysis present in Section 2.3. At the smallest value of elliptical error, the measured nonlinear noise is within a factor of three above the simulation, which can be attributed to the presence of other nonlinearities, particularly the nonelliptical nonlinearity laid out in Section 2.4 and the residual nonlinearity of the coil-magnet drive. The baseline noise curve shows the sum of all measured noises in the quiet (‘zero-displacement’) state and represents the absolute noise floor of the sensor. This sensitivity is significantly above the noise performance we demonstrated in Ref. [8], but this can be explained by three main factors: (i) the setup only includes a single sensor, meaning there is no frequency stabilisation; (ii) the box is not as well insulated with packing foam, meaning air currents are not as suppressed; and (iii) the rubber pad necessary to enable differential driving of the mirror and sensor also breaks the common mode rejection of seismic noise present in the original null measurement.

Figure 5 shows the result of deliberately inducing nonlinearities through varying ellipticity. The nonlinear noise is matched well by our time-domain simulation, particularly for large values of the elliptical error (δ). The difference between the measured and simulated noise for the smallest δ value can be explained through the presence of other nonlinearities, which begin to dominate at small values of δ. While some of this discrepancy can be attributed to the residual nonlinearity of the drive, it can be entirely explained by the limiting nonelliptical nonlinearity discussed in Section 2.4, which we show to have a similar effective contribution as a δ of 2%. In this scheme, we are operating sufficiently below the velocity limit of imposed in Section 2.5, which means this source of nonlinearity should not limit the readout.

## 4. Sensitivity Projections in the LIGO Vacuum Chambers

We have demonstrated the loss of sensitivity due to nonlinearities in a carefully contrived scenario. However, any scenario that requires access to the full proposed dynamic range of the sensor may encounter problems with the sensor linearity. We mentioned the application in inertial sensors, which is highly relevant to the field of GW detection. An even closer application is in the sensing of the multistage suspension systems that are found in all contemporary GW detectors. In this section, we take the example of the Advanced LIGO quadruple pendulum suspensions [9].

The suspensions in the Advanced LIGO chambers are already placed within a seismically isolated environment on top of the so-called internal seismic isolation (ISI). The suspension stages provide progressively greater levels of vibration filtering, which means their motion is, over many frequencies, even smaller than the residual motion injected by the ISI. Therefore, this application is intuitively less susceptible to nonlinearities due to its low RMS displacement.

We consider, specifically, the measurement of longitudinal displacement sensing of the top mass of the quadruple chain. This is the location of the sensing and control of most of the 24 suspension degrees of freedom and the location of the majority of the existing displacement sensors. We propose our device as a candidate to precisely replace these sensors in order to achieve the required 100 factor of improvement in the suspension sensing noise, as laid out in Ref. [11].

**Figure 6:**
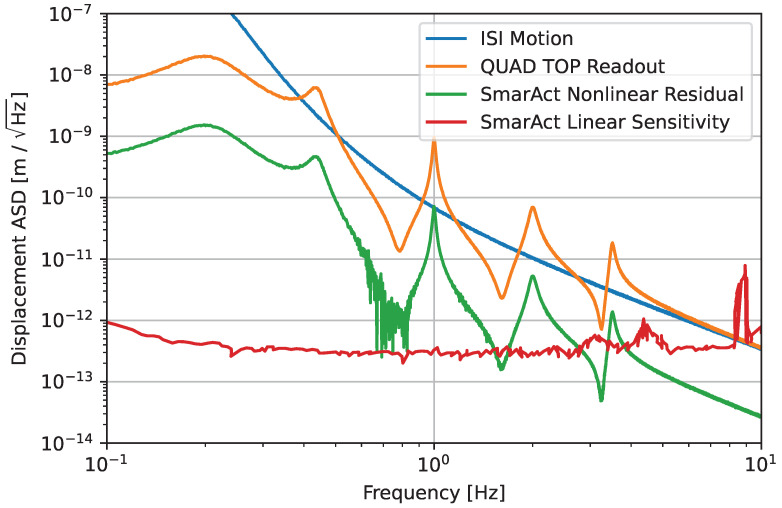
Simulated readout of the top stage of the LIGO quadruple suspension with the SmarAct sensor, together with the corresponding residual nonlinear error. The ISI motion shows the inertial displacement of the ISI, and the QUAD TOP readout represents the displacement of the top quadruple suspension stage as measured by an ideal sensor rigidly attached to the ISI (i.e., the relative displacement between the top stage and ISI). While the residual shows that nonlinear noise significantly exceeds the nominal noise floor of the device, at no point in the spectrum does the nonlinear residual exceed the measured signal. The SmarAct sensitivity derived from a null measurement is added for comparison.

We generate an ISI noise model based on the measured ISI displacement spectral density. We further introduce a fit to the ISI horizontal-to-top mass horizontal transfer function in order to estimate the representative relative displacement spectrum between the top mass and ISI. We pass this displacement spectrum through our model of the sensor nonlinearity, assuming a nominal elliptical error of 2%. As found in our investigations above, it is possible to reach a hard limit of the nonelliptical nonlinearities at this level. The simulated displacement spectrum with the corresponding nonlinear noise level is shown in Figure 6. As shown, the nonlinear noise can be significantly higher than the quoted sensitivity during a null measurement, specifically when the true displacement is also high. However, the spectral density of the nonlinearity clearly shadows the spectral density of the signal at a level that is around a factor of 10 or more below across the whole frequency band of interest. This seemingly linear scaling of this additional noise with signal amplitude is due to the nonlinear effect leading to a beat between components at different frequencies. In this case, the beat between the signals across the frequency band and the broad, dominant peak at 0.2 Hz is the cause of the nonlinear error.

## 5. Conclusions

Interferometric displacement sensors are poised to replace many existing electromagnetic and optical sensing schemes within applications requiring subpicometre levels of sensitivity. In this paper, we follow-up the investigation of our custom sensor manufactured by SmarAct reported in Ref. [8], with a focus on the sensor’s linearity. We embed this investigation within the specific context of inertial sensing and gravitational wave detection. The former use case is motivated by the sensor’s future implementation on the compact six-degrees-of-freedom inertial sensor prototyped in Ref. [21]. The latter is in recognition of the sensor’s suitability as a future candidate sensor on the upgraded Advanced LIGO quadruple suspensions [9].

We briefly lay out the key equations that describe the deep frequency modulation technique that we employ within our readout scheme. From this starting point, we show the possible origins of the nonlinear couplings to the sensor readout and analyse their impact on the displacement sensitivity. We find that an imprecise fit (or drift) of the ellipticity of the Lissajous figure constructed from the two orthogonal readout signals is often the dominant source of nonlinear noise for elliptical error in excess of 2%. We subsequently revisit our measurement of the current hard limit to the nonlinearity, which is generated by periodic error due to distortion of the Lissajous figure, which cannot be corrected in real time or in post processing. This leads to an effective elliptical error of 2%.

We construct a band-limited, high-RMS scenario in which the nonlinear error can significantly exceed both the linear noise level and the true displacement spectrum at low frequencies. We conduct an experimental demonstration of this nonlinear noise and compare the results to a time-domain simulation, which show good agreement with each other. We also estimate an order-of-magnitude figure for the maximum nonlinear noise level in the presence of a broadband signal and find that it is consistent with measurement.

Overall, the linearity of the sensor is something that should be carefully considered based on the precise parameters of a given application, particularly the RMS displacement and the required dynamic range. We find that the nonlinear noise may limit the sensitivity of inertial sensors if not managed well. However, we find that on relatively quiet platforms, such as the Advanced LIGO ISI, the linearity of the sensor is sufficient to ensure an SNR above unity within the detection band; thus, no significant improvements to the sensor performance are necessary.

Despite the generally sufficient linearity of the sensor for applications at the heart of our investigation, there is certainly scope for further improvement of the sensor’s linearity. Future experimental work on the sensor we presented herein will focus on implementing more advanced algorithms that seek to improve long-term stability and, potentially, real-time correction and linearisation of the system.

## Abbreviations

The following abbreviations are used in this manuscript:

BOSEM	Birmingham Optical Sensor and Electromagnetic Motor
BRS	Beam rotation sensor
CDS	Control and design system
DFM	Deep frequency modulation
FPGA	Field-programmable gate array
FSR	Free spectral range
GW	Gravitational wave
HoQI	Homodyne quadrature interferometer
ISI	Internal seismic isolation
LIGO	Laser Interferometer Gravitational-Wave Observatory
LISA	Laser interferometer space antenna
PSD	Power spectral density
PZT	Piezoelectric transducer
RMS	Root mean square
SNR	Signal-to-noise ratio
